# Chronic Atrophic Gastritis Presenting as Hemolytic Anemia due to Severe Vitamin B12 Deficiency

**DOI:** 10.1155/2021/9571072

**Published:** 2021-07-31

**Authors:** Amanda M. Woodford, Rabhea Chaudhry, Gabriella A. Conte, Varsha Gupta, Madhurima Anne

**Affiliations:** ^1^Department of Medicine, Jersey Shore University Medical Center, Hackensack Meridian Health, Neptune Township, NJ, USA; ^2^Department of Hematology and Oncology, Jersey Shore University Medical Center, Hackensack Meridian Health, Neptune Township, NJ, USA

## Abstract

Vitamin B12 is an essential nutrient which plays an important role in neurological function, hematopoiesis, and DNA synthesis. Low levels usually stem from either poor intake or a malabsorptive process. Presently, the most common cause of vitamin B12 deficiency is food-bound cobalamin malabsorption, which occurs when there is impaired release of vitamin B12 from ingested food due to an outstanding factor preventing the release of the nutrient from its transport protein. Such causes include achlorhydria, gastritis, gastrectomy, or the use of PPIs or antacids. A rarer cause is autoimmune chronic atrophic gastritis, resulting in pernicious anemia. In this disease process, there is destruction of parietal cells and thus a reduction in intrinsic factor, which is essential to the absorption of vitamin B12. Deficiency will result in a variety of abnormalities including but not limited to pancytopenia, paresthesias, and neuropsychiatric symptoms. A rare manifestation of vitamin B12 deficiency is hemolytic anemia, which occurs due to intramedullary and extramedullary dysfunction. This case describes a 46-year-old male with no past medical history who presented with chest pain, fatigue, and progressive weakness, found to have hemolytic anemia, ultimately attributed to vitamin B12 deficiency. Antiparietal cell antibodies and intrinsic factor antibodies (IFA) were both negative. Still, the patient underwent an endoscopy with biopsies of the stomach; pathology was consistent with chronic metaplastic atrophic gastritis. The patient improved with intramuscular vitamin B12 supplementation. This case highlights both a rare cause and presentation of vitamin B12 deficiency. Patients with autoimmune chronic atrophic gastritis should have antiparietal cell or intrinsic factor antibodies. Still, seronegative patients have been reported, like this patient. Additionally, hemolytic anemia secondary to vitamin B12 deficiency is uncommon. The presentation will usually mirror that of a thrombotic microangiopathy (TMA), including hemolytic anemia with schistocytes on peripheral blood smear and thrombocytopenia, as it did in this patient. This clinical entity is described as pseudothrombotic microangiopathy and is crucial to identify in order to prevent the initiation of invasive treatment strategies such as plasmapheresis.

## 1. Introduction

Vitamin B12 (cobalamin) is an essential nutrient that acts as a cofactor for major chemical reactions in the body, including the conversion of methylmalonic acid to succinyl coenzyme A, the conversion of homocysteine to methionine, and the conversion of 5-methyltetrahydrofolate to tetrahydrofolate, which are crucial to proper neurologic function, hematopoiesis, and DNA synthesis [1]. Consequently, deficiency will usually manifest as a compilation of hematologic irregularities (macrocytic anemia, thrombocytopenia, or pancytopenia), neurologic symptoms (paresthesias, loss of proprioception, or autonomic nervous system dysfunction), and/or as neuropsychiatric abnormalities such as depression, dementia, or mania [2]. The prevalence of vitamin B12 deficiency in the United States has been reported to be as high as 3% in those aged 20–39 years old, 4% in those aged 40–59 years old, and 6% in those over 60 years old [3]. A lesser-known complication of vitamin B12 deficiency is hemolytic anemia, sometimes presenting as a pseudothrombotic microangiopathy. This can occur due to both intramedullary and extramedullary dysfunction. Deficiency leads to ineffective erythropoiesis, resulting in intramedullary hemolysis and the release of lactate dehydrogenase (LDH), in addition to impaired deformability of the red blood cell membrane, which causes extramedullary fragmentation and hemolysis [2, 4].

Mainly obtained through the ingestion of fish, meat, dairy products, and fortified cereals, vitamin B12 is absorbed in the terminal ileum along with intrinsic factor, which is produced by parietal cells in the stomach [1]. Deficiency usually results from either poor intake or a malabsorptive process. The most common etiology of vitamin B12 deficiency is food-bound cobalamin malabsorption, which occurs when there is impaired release of vitamin B12 from ingested food due to an outstanding factor that prevents the release of the nutrient from its transport protein. Such causes include achlorhydria, gastritis, gastrectomy, or the use of proton-pump inhibitors (PPIs) or antacids [3]. A rarer cause of vitamin B12 deficiency is autoimmune chronic atrophic gastritis, leading to pernicious anemia. In this disease, there is an underlying inflammatory process which leads to the destruction of parietal cells and thus a reduction in intrinsic factor, which is essential to the absorption of vitamin B12. Diagnosis is usually made via positive serologic studies, such as the presence of anti-intrinsic factor antibodies or antiparietal cell antibodies, or through histological examination [5]. Herein, we present a case of vitamin B12 deficiency manifesting as hemolytic anemia secondary to chronic autoimmune gastritis as observed on histologic examination, although with negative serologic markers.

## 2. Case Report

A 46-year-old Haitian male with no past medical history presented to the emergency department with chest pain, progressive weakness, and fatigue that had been ongoing for the past month. Prior to these symptoms, the patient was in his usual state of health. He did not routinely follow up with a medical provider. He described the chest pain as an intermittent, substernal burning sensation that occurred mainly at rest. It caused him to have decreased appetite, resulting in an unintentional 30lb weight loss over the preceding several weeks. He denied fevers, chills, cough, night sweats, dysphagia, odynophagia, nausea, or vomiting. He also reported constipation, with bowel movements every 3-4 days associated with occasional rectal pain and bright red blood with wiping. He otherwise denied any rectal bleeding, hematochezia, melena, hematemesis, hematuria, rashes, or bruising. Of note, the patient had no significant family medical history. He took no medications and denied use of over-the-counter agents including NSAIDs, acetaminophen, aspirin, herbs, or supplements. He denied alcohol or tobacco use. He consumed a diet including meat and vegetables with no restrictions. He reported working outside as a landscaper, although denied any known tick or mosquito bites. He denied any recent sick contacts or travel history, with his last trip to Haiti occurring about 1.5 years prior to presentation.

The patient's vital signs on admission were as follows: body temperature 98.1°F, pulse of 84/min, respiratory rate of 18/min, BP 130/67 mmHg, and oxygen saturation of 100% on room air. Physical exam was notable for scleral icterus. There were no oral ulcers or glossitis. The abdomen was soft with no tenderness or hepatosplenomegaly. There was no peripheral edema or rashes. Neurologic examination was unremarkable; muscle strength was 5/5 in the upper and lower extremities, and there was no sensory deficit exhibited. He also had no gait abnormalities.

Initial complete blood count (CBC) revealed pancytopenia with a decreased white blood cell count of 2.4uL (4.5–11.0 10 *∗* 3/uL), hemoglobin level of 4.9 g/dL (13.2–17.5 g/dL), and platelet count of 5710 *∗* 3/uL (140–450 10 *∗* 3/uL). Mean corpuscular volume was 98.7 fL (80.0–100.0 fL). Absolute reticulocyte count was 0.020 × 10^6/uL (0.010–0.110 × 10^6/uL). Peripheral blood smear was grossly abnormal, demonstrating tear drop cells, few schistocytes, anisopoikilocytosis, left shifted white cells, giant platelets, atypical lymphocytes, and no blasts. Additional blood work revealed normal serum electrolytes and kidney function, an elevation of AST, total bilirubin, direct bilirubin, and LDH, and a decreased haptoglobin level (Table 1). A direct Coombs IgG test was positive. Vitamin B12, folate, and homocysteine levels were also drawn at this time as part of the workup for hemolytic anemia, which demonstrated severe vitamin B12 deficiency (<50 pg/mL), normal folate levels of 22 (>4.0 ng/mL), and elevated homocysteine level of 42.6 (5.0–15.0 umol/L). Urinalysis showed hazy urine, negative for glucose, bilirubin, and ketones, moderate blood, 30–50 RBCs, and 0–2 WBCs. CT scan of the chest/abdomen/pelvis with contrast was significant for mild splenomegaly with the spleen measuring 13.9 cm in length, with no radiographic evidence of splenic infarcts or lesions. There was no hepatomegaly or significant retroperitoneal or mediastinal lymphadenopathy. The stool occult test was positive for occult blood. The patient was transfused 2 units of packed red blood cells and admitted for further workup.

Additional testing was performed to evaluate for hemolytic anemia, including hepatitis panel, HIV, CMV, EBV IgM, ANA, and tick-borne pathogens including malaria/*Babesia* parasite smear, *Lyme* antibodies, *Ehrlichia* antibodies, *Rickettsia rickettsii* antibodies, and ADAMTS13 levels. While the results of these tests were not available on the day of admission, all ultimately came back negative or within normal limits. Peripheral blood flow cytometry showed no immunophenotypic evidence of lymphoproliferative disorder, high-grade myeloid neoplasm/acute leukemia, or plasma cell neoplasm.

**Figure 1:**
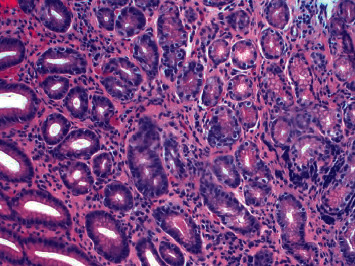
Gastric mucosa demonstrating chronic gastritis, intestinal metaplasia identified by Alcian blue-PAS stain, and absence of oxyntic glandular tissue, consistent with metaplastic atrophic gastritis. No dysplasia identified. No *Helicobacter pylori* identified by Giemsa stain.

Thrombotic thrombocytopenic purpura was initially considered, but less likely given the patient was afebrile, had normal mentation and normal kidney function. ADAMTS13 eventually resulted within normal limits. The leading hypothesis for his pancytopenia and hemolytic anemia was ineffective erythropoiesis and intramedullary hemolysis from severe vitamin B12 deficiency. Antiparietal cell antibodies and intrinsic factor antibodies (IFA) were sent; both ultimately resulted negative. The patient subsequently underwent upper endoscopy (EGD) and colonoscopy. EGD revealed a single, nonbleeding erosion at the gastroesophageal junction and diffuse atrophic appearing mucosa in the gastric body and antrum, which was biopsied. Pathology demonstrated gastric antrum mucosa with chronic gastritis and no intestinal metaplasia or *Helicobacter pylori* identified; gastric mucosa was consistent with chronic gastritis, intestinal metaplasia, and an absence of oxyntic glandular tissues, consistent with metaplastic atrophic gastritis (Figure 1). Colonoscopy showed internal hemorrhoids but was otherwise grossly unremarkable.

The day following admission, the patient's posttransfusion hemoglobin improved to 8.2 g/dL (13.2–17.5 g/dL). He was ultimately treated with 4 days of intravenous dexamethasone 40 mg daily for possible autoimmune hemolytic anemia, given the initial direct positive Coombs test, and was started on daily intramuscular injections of cyanocobalamin 1000 mcq for a total of 10 days. He did not require further blood transfusions. Through the course of his hospitalization, he continued to improve, reporting increased energy and better appetite. On the day of discharge, the patient's hemoglobin was 9.8 g/dL (13.2–17.5 g/dL). Given that the patient's cell counts showed improvement with vitamin B12 supplementation, bone marrow biopsy was deferred. He was discharged on oral vitamin B12 supplementation and instructions to follow up in the hematology clinic to receive weekly intramuscular vitamin B12 injections for the following month.

## 3. Discussion

Vitamin B12 deficiency presenting with severe hematologic manifestations is a relatively uncommon occurrence, with only a limited number of reports in the current medical literature. Our case not only serves as an example of this rare clinical entity but also presents a constellation of unique findings of this particular disease process. First, our patient's initial presentation included hemolytic anemia, thrombocytopenia, and dysmorphic red blood cells. These findings are consistent with a thrombotic microangiopathy (TMA). However, because vitamin B12 levels were obtained immediately and were shown to be significantly decreased, the diagnosis of pseudo-TMA, a TMA specifically secondary to vitamin B12 deficiency, was made. Unlike primary TMA syndromes, such as thrombotic thrombocytopenic purpura or hemolytic uremic syndrome, pseudo-TMA does not require invasive interventions like plasmapheresis. It is speculated that the etiology of dysmorphic red blood cells in severe vitamin B12 deficiency stems from both intramedullary and extramedullary dysfunction. From an extramedullary perspective, research has shown that hyperhomocysteinemia, which occurs in vitamin B12 deficiency, may increase the risk of hemolysis in addition to causing endothelial damage, which further leads to intravascular hemolysis and RBC fragmentation [6]. Of note, hyperhomocysteinemia can also be caused by hypothyroidism, obesity, diabetes, hyperlipidemia, medications, and more rarely possessing a methylenetetrahydrofolate reductase gene variant [7]. Testing of this gene variant though is usually only recommended if the cause of the hyperhomocysteinemia is unexplained by another more common cause, thus why our patient did not undergo this form of testing.

Additionally, as more data were accumulated, the suspected cause of our patient's vitamin B12 deficiency was autoimmune atrophic chronic gastritis, given the histologic findings noted on biopsies of his stomach, specifically the intestinal metaplasia and absence of oxyntic glandular tissues in the gastric mucosa, no evidence of *Helicobacter pylori* infection, and lack of metaplasia in the gastric antrum. According to the Updated Sydney System of Classification and Grading of Gastritis, autoimmune gastritis is strongly associated with corpus predominant atrophic gastritis, with little to no atrophy or metaplasia noted in the antrum [8]. This would imply the patient had pernicious anemia, leading to severe vitamin B12 deficiency and thus hemolytic anemia. Although rare, reports of this disease process exist in the literature. However, in a majority of these cases, the diagnosis of pernicious anemia is demonstrated through histological exam in addition to laboratory studies, or evidence of prior gastrectomy or ileum resection [9–11]. Historically, these laboratory studies included surveilling for intrinsic factor deficiency using the Schilling test [12]. However, this method is frequently being replaced by other diagnostics tests such as detecting intrinsic factor antibodies or parietal cell antibodies. Although intrinsic factor or parietal cell antibodies were not detected in our patient, he had histologic evidence of autoimmune gastritis confirming the diagnosis.

At the time of this publication, only a few cases were found which described a patient presenting with hemolytic anemia due to severe vitamin B12 deficiency, likely secondary to seronegative chronic atrophic gastritis. Similar to our patient, this case describes a young male presenting with a macrocytic anemia, elevated LDH, and undetectable vitamin B12 levels, who was discovered to have chronic atrophic gastritis via biopsy. This patient also had negative serologic markers, including parietal cell antibodies and intrinsic factor antibodies [13].

Recently, Conti et al. performed a retrospective, cross-sectional study in Italy examining levels of antiparietal cell antibodies (PCAs) and clinical features of 516 patients diagnosed with autoimmune atrophic gastritis by histology. Of these patients, 21.1% resulted seronegative. The seronegative patients were significantly older than the seropositive patients, with a greater proportion of these patients over the age of 70. It was speculated that the positive correlation between older age and seronegativity could be explained by a seroconversion due to progression of disease, similar to what was observed in other autoimmune conditions, such as autoimmune thyroiditis. However, this study found that seropositivity did not correlate with atrophy severity and that seronegativity was not associated with greater severity of gastric atrophy or clinical symptoms [14]. Interestingly, our case presents a young patient with negative serologic studies.

Lastly, another unique feature of our case was the presence of an initial positive direct antiglobulin test (DAT) in the setting of hemolytic anemia caused by vitamin B12 deficiency. Pernicious anemia associated with a positive DAT has been rarely described in the literature. It has been reported that patients with pernicious anemia may transiently have a positive Coombs test; however, repeat testing will often become negative after that patient is started on vitamin B12 supplementation [15]. Interestingly, in our patient, repeat DAT was negative after the patient had been started on both vitamin B12 supplementation and a course of steroids.

## 4. Conclusion

This case highlights both a rare cause and manifestation of vitamin B12 deficiency. Deficiency occurs due to poor oral intake or a malabsorptive process of the nutrient. Autoimmune chronic atrophic gastritis is a rare etiology of vitamin B12 deficiency, which results in a destruction of the parietal cells, leading to a reduction in intrinsic factor, which is essential to the absorption of vitamin B12. In this disease process, patients will usual have either antiparietal cell or intrinsic factor antibodies. However, recent studies have demonstrated patients with this disease who are seronegative, making biopsy essential to diagnosis. Additionally, vitamin B12 deficiency usually presents with neurologic and hematologic symptoms. Rarely, it will cause hemolytic anemic and thrombocytopenia, best described as a pseudothrombotic microangiopathy, as demonstrated in this case. It is speculated that the mechanism of hemolysis is due to both intramedullary and extramedullary dysfunction. It is critical to establish this diagnosis early on to prevent unnecessary treatment, such as plasmapheresis.

## Figures and Tables

**Table 1:** Laboratory data.

Lab	Value	Reference range
WBC	2.4	4.5–11.0 10 *∗* 3/uL
ANC	1.2	1.8–8.0 10 *∗* 3/uL
HGB	4.9	13.2–17.5 g/dL
HCT	14.9	40.0–53.0%
MCV	98.7	80.0–100.0 fL
Platelet	57	140–450 10 *∗* 3/uL
Glucose	93	70–99 mg/dL
Na	138	136–145 mmol/L
K	4.4	3.5–5.2 mmol/L
Chloride	105	96–110 mmol/L
Carbon dioxide	21	24–31 mmol/L
BUN	14	5–25 mg/dL
Cr	1.15	0.61–1.24 mg/dL
AST	105	10–42 U/L
ALT	55	10–60 U/L
ALP	29	38–126 U/L
Total bilirubin	4.2	0.2–1.3 mg/dL
Direct bilirubin	0.5	0.0–0.2 mg/dL
Troponin	0.01	<0.08 ng/mL
LDH	6392	91–200 U/L
Haptoglobin	<6	30–225 mg/dL
Reticulocyte count	1.38	0.40–2.50%
Direct coombs IgG	Positive	Negative
Ferritin	304.3	24–336 ng/mL
Serum iron	81	45–180 ug/dL
TIBC	227	260–480 ug/dL
Vitamin B12	<50	180–914 pg/mL
Folate	22	>4.0 ng/mL
Homocysteine	42.6	5.0–15.0 umol/L
Intrinsic factor antibody	Negative	Negative
Antiparietal cell	20.7	0.0–20.0 units = negative
Antibodies (first test)		20.1–24.9 = Equivocal
Antiparietal cell	19.2	0.0–20.0 units = negative
Antibodies (second test)		20.1–24.9 = Equivocal

## References

[1] Langan R. C., Goodbred A. J. (2017). Vitamin B12 deficiency: recognition and management. *American Family Physician*.

[2] Stabler S. P. (2013). Vitamin B12 deficiency. *The New England Journal of Medicine*.

[3] Shipton M. J., Thachil J. (2015). Vitamin B12 deficiency-A 21st century perspective. *Clinical Medicine*.

[4] Sasi S., Yassin M. A. (2020). A rare case of acquired hemolytic anemia and pancytopenia secondary to pernicious anemia. *Case Reports in Oncology*.

[5] Rodriguez-Castro K. I., Franceschi M., Noto A. (2018). Clinical manifestations of chronic atrophic gastritis. *Acta Biomed*.

[6] Fahmawi Y., Campos Y., Khushman M. d. (2019). Vitamin B12 deficiency presenting as pseudo-thrombotic microangiopathy: a case report and literature review. *Clinical Pharmacology: Advances and Applications*.

[7] U.S. Department of Health and Human Services. MTHFR gene variant. Genetic and Rare Diseases Information Center, https://rarediseases.info.nih.gov/diseases/10953/mthfr-gene-variant

[8] Dixon M. F., Genta R. M., Yardley J. H., Correa P. (1996). Classification and grading of gastritis. *The American Journal of Surgical Pathology*.

[9] Veit K. (2017). Pseudothrombotic microangiopathy and vitamin B12 deficiency in pernicious anemia. *Baylor University Medical Center Proceedings*.

[10] Osman H., Alwasaidi T. A., Al-Hebshi A., Almutairi N., Eltabbakh H. (2021). Vitamin B12 deficiency presenting with microangiopathic hemolytic anemia. *Cureus*.

[11] Harada Y., Komori I., Morinaga K., Shimizu T. (2018). Microangiopathic haemolytic anaemia with thrombocytopenia induced by vitamin B12 deficiency long term after gastrectomy. *BMJ Case Reports*.

[12] Rodriguez N. M., Shackelford K. (2021). Pernicious anemia. *StatPearls*.

[13] Cittolin-Santos G. F., Khalil S., Bakos J. K., Baker K. (2020). Chronic atrophic gastritis with negative intrinsic factor and parietal cell antibody presenting as a severe hemolytic anemia. *Case Reports in Hematology*.

[14] Conti L., Lenti M. V., Di Sabatino A. (2020). Seronegative autoimmune atrophic gastritis is more common in elderly patients. *Digestive and Liver Disease*.

[15] Yeruva S. L. H., Manchandani R. P., Oneal P. (2016). Pernicious anemia with autoimmune hemolytic anemia: a case report and literature review. *Case Reports in Hematology*.

